# Perceptions of Waterpipe Smoking among Young Adults: A Phenomenological Study

**DOI:** 10.3390/dj8040134

**Published:** 2020-12-10

**Authors:** Amraj. Z. Dhillon, Tim Doran, Vishal. R. Aggarwal

**Affiliations:** 1 Heart Centre, The William Harvey Research Institute, Queen Mary University of London, Charterhouse Square, London EC1M 6BQ, UK; 2 Department of Health Sciences, University of York, York YO10 5DD, UK; tim.doran@york.ac.uk; 3 School of Dentistry, University of Leeds, Clarendon Way, Leeds LS2 9JT, UK

**Keywords:** waterpipe, tobacco, smoking, qualitative, young adults, perceptions

## Abstract

Background: Waterpipe tobacco smoking is becoming increasingly popular in the West among young people. Given the associated health risks of this behaviour, we aimed to investigate why young adults take up waterpipe tobacco smoking and their attitudes to the associated risks. Methods: This was a qualitative study, with a phenomenological perspective. Focus groups and face-to-face semi-structured interviews were conducted among waterpipe smokers aged 20–30 years living in London, UK. Transcripts were analysed using constant comparison and cycling between the data and analysis. Data collection continued until thematic saturation was achieved. Results: Sixteen smokers attended focus groups and face-to-face interviews. Seven main themes emerged encompassing four main dimensions relating to: lack of knowledge, perceived risk, affordability, and social addiction. Waterpipe was perceived to be safer than cigarette smoking due to the pleasant odour, fruity flavours, and belief that water filtered the toxins. The waterpipe had become a “social addiction” enhancing group atmosphere, was cheap and did not have the dangers of violence or hallucinations associated with other addictions like alcohol and drugs. Because of their intermittent smoking patterns and the lack of statutory warnings, none felt they were not at risk of any adverse side effects. Conclusions: Waterpipe smoking is a growing public health problem; the social environment associated with its use is perceived to be a positive outlet by smokers who do not consider it harmful to their health. These perceptions need to be addressed urgently by anti-smoking policies.

## 1. Implications

- There has been an explosion of waterpipe usage and its social acceptance among young adults.
- We found that lack of knowledge, perceived risk, affordability, and social addiction have all contributed to increased use and social acceptance.
- Accurate and targeted prevention is important, which takes into consideration the strong social standing, cultural motives, and adverse health effects associated with waterpipe tobacco smoking.

## 2. Introduction

In 2015, the World Health Organization advised that waterpipe tobacco smoking (WTS) is detrimental to health and it is not a safer form of tobacco use than cigarette smoking [1]. Despite this, global statistics indicate increased use [2], particularly in school and university settings, where many people from the Western World first acquire the habit [3]. Within the United Kingdom [UK], it is estimated that 66% have tried WTS and 8.7% smoke at least once monthly [4].

A growing body of published evidence indicates that WTS carries a similar risk to health as cigarette smoking [5]. Waterpipe smoking involves tobacco, but is less regulated than cigarettes and other tobacco-based products and in many cases there is a higher nicotine content. Components of waterpipe smoke, such as volatile aldehydes, hydrocarbons, and carbon monoxide [6,7], can lead to the development of malignancy and respiratory disease [5,8]. The use of shared oral tubes is also associated with transmission of infectious diseases [9] including respiratory infections [10] and human papillomavirus (HPV) [11]. Despite this, over 70% of current waterpipe smoker’s believe that it is less harmful than cigarettes [3,12,13,14,15,16,17]. Attitude, beliefs and perceptions of WTS are multifactorial with culture and a lack of public health initiatives playing a key role [18]. A common misconception is that hazardous substances are filtered from the smoke as it passes through the water [18,19,20], but current evidence does not support this perception [21].

The increasing uptake of WTS is a growing public health concern, particularly among young British adults [22]. Previous research has explored the university setting [3,14,23], providing predominantly quantitative insights into smoking habits and general perceptions. Roskin et al. found that students felt WTS might be something they would not continue after university [23], thus it is plausible that graduates may demonstrate different behaviours and attitudes to students. Unlike university students, young British graduates and working professionals who engage with WTS outside the university setting may continue this habit, as it becomes part of their social routine. If appropriate prevention and cessation strategies, policies and interventions are to be formulated, it is important to understand the cultural, emotional, social and other factors that influence uptake of waterpipe smoking as well as perceptions of health risk compared to other types of tobacco consumption [24]. This study therefore aims to investigate why young adults smoke waterpipe tobacco and their attitudes on the associated risks. Specific objectives are to

- investigate reasons for the growing popularity of WTS among young graduates,
- investigate their attitudes towards the health risks associated with this habit, and
- gain a deeper understanding of the social environment surrounding WTS.

## 3. Methods

### 3.1. Study Design

The study design was a qualitative study that used a phenomenological approach so as to explore the individual’s experience of WTS and aim to see the “reality” or “truth” of this experience in the “eyes” of the smokers [25].

#### 3.1.1. Participants and Sampling

We used a purposive sample to ensure that a variety of smoking habits and frequencies were covered. The target population was people 20 to 30 years of age who had graduated and/or were in employment and smoked the waterpipe a minimum of once a month—many acquire the habit while in education and the transition from the university environment to the real world that is of interest, as the social pressures and habits are very different between the two groups. Given that the use of licit and illicit substances plays an important role in many university students’ lives [26] and young adult behaviour is likely a foundation for lifelong health trajectories [27], understanding why this cohort continues to use WTS after leaving higher education is important so that prevention strategies can be targeted at this group of early smokers and mitigate future morbidity and mortality from harmful effects of WTS.

Smokers were identified in London Shisha cafés and asked to complete a questionnaire (Supplementary Figure S1). As well as experiencing the greatest growth in Shisha cafés [28], London is a cosmopolitan and diverse city with many graduates and young professionals, and results are likely to be generalisable to other large cities in the UK and global metropoles. The questionnaire allowed for identification of waterpipe smokers within the desired age range (purposive sampling) and ensured that a wide variety of smoking frequencies (i.e., heavy and light smokers) and habits (e.g., cigarette smokers and non-cigarette smokers) were recruited for the study. The questionnaire was adapted from the Global Tobacco Surveillance System, Tobacco Questions for Surveys by the World Health Organization 2011 [29]. Both genders were targeted to ensure that the study also explored gender differences (if any) in perceptions of WTS.

Waterpipe smokers identified in cafés were also asked if they were aware of any frequent “home smokers”.

Suitable subjects were invited to participate using study information sheets explaining the nature of the research. Prior to each interview, participants were verbally informed about the study and asked to sign an informed consent form.

#### 3.1.2. Ethical Approval

The Queen Mary Ethics Research Committee granted ethical approval for this study (QMREC2013/21).

#### 3.1.3. Data Collection

Data collection was guided by an interpretative phenomenological approach [30]. Recruitment was two-fold: (1) semi-structured focus groups of 4–6 smokers and (2) one-to-one in-depth interviews with participants who had not taken part in the focus groups. Interviews took place in Shisha Café’s or the Queen Mary Dental Institute; whichever setting was most convenient for the participants. The former setting is where a smoker is likely to feel most comfortable discussing waterpipe smoking, therefore allowing free expression, whereas the latter setting took the participants out of their usual smoking environment, which may have allowed them to assess their own behaviour from a different perspective. Two focus groups and nine in-depth interviews were conducted, with an average length of 84 min and 58 min respectively.

We developed a topic list to guide interviews (Supplementary Figure S2) following the review of the literature [13,23,24,31]. This was further developed in parallel with the analysis, using a constant comparative technique [32]. Topics included experiences and understanding of the waterpipe, views on smoking, smoking habits, motivations for smoking and levels, acceptability and perceptions on health risks. The interviewer, a non-smoker, adopted a participant-observer role. The researcher maintained a journal to allow them to reflect on their interpretation of the interviews, which formed an important part of the audit trail to increase transparency [30].

The number of interviews conducted and the size of the sample were sufficient to identify themes from this group of waterpipe smokers. No new themes or data emerged from later interviews, with thematic saturation being reached.

#### 3.1.4. Data Analysis

Recordings were transcribed ad verbatim. Transcriptions were checked for accuracy and context by the interviewer and two transcripts were given to the interviewees to read to allow respondent validation.

Data analysis took the form of thematic content analysis. The development of the coding framework was informed by a “bottom-up” approach. Thereafter, the code was applied systematically to the transcripts.

## 4. Results

### 4.1. Response Rates and Demographics of Participants

Sixteen participants were identified from three cafes located in three different London Boroughs. We conducted two focus groups (one group of four and another group of three) and nine in-depth face-to-face interviews in April 2014. In both focus groups, there was a “no-show” of two participants.

There was a diverse range of ethnic backgrounds, with females more willing to participate (63%) (Table 1). All of those interviewed had graduated from university, with most working in the city of London. The majority began WTS at university (88%), smoked mainly at café’s (81%) and did not use any other form of tobacco (88%) (Table 1).

### 4.2. Thematic Analysis

We extracted the following key themes that encompassed four main dimensions related to lack of knowledge, perceived risk, affordability, and social addiction that may influence the intention to smoke:

#### 4.2.1. Availability & Affordability

Respondents claimed the waterpipe has become readily available (*n* = 11), which encouraged uptake. The proximity of a café to a smoker’s home played a key role in frequency of use, with no respondents willing to travel large distances to smoke. Waterpipe smoking was also seen as an affordable way to socialise with friends (*n* = 7), as one waterpipe is often shared among a group of friends, thereby dividing the cost (Table 2).

#### 4.2.2. Social Addiction

- Social appeal

Most smoked exclusively in Shisha cafés (*n* = 13), with none smoking exclusively at home. The number of “social gatherings” and occasions was a determining factor on the frequency of WTS. It was considered to be a fashionable “on-trend” group activity. Some were amused at the idea of smoking alone, especially in a café setting (*n* = 3). The waterpipe allowed those that do not drink alcohol (*n* = 8), due largely to cultural and religious reasons, to take part in a social experience. Comparisons were made to social drinking (*n* = 10), rather than other smoked tobacco products such as cigarettes (Table 3).

- Environment

The atmosphere and surroundings provided by Shisha cafés emerged as a dominant theme (*n* = 13). Several respondents did question whether it was the waterpipe itself they truly enjoyed smoking or the environment in which they smoke. Opinions were divided as to whether their smoking frequency decreased during winter. With the right social circle, many felt cold weather only played a small part in decisions to smoke waterpipes at a café. There was also mention of an “underground” culture of indoor Shisha cafés, despite the UK’s smoking bans (*n* = 3). (Table 3)

- Cultural Relevance & Family Acceptance

Culture and ethnic backgrounds surfaced as a major theme (*n* = 11). Respondents expressed the link to their heritage (Asian or Arabic) and how the waterpipe is an alternative to alcohol, which for some is forbidden by their religion. Those who also smoked at home (*n* = 3) expressed a stronger cultural link to the waterpipe. Those with no direct cultural link to waterpipe use stated that they tend to smoke more frequently with their Asian and Middle Eastern friends. Some reported being open with their parents about waterpipe smoking (*n* = 3), but were aware that cigarette smoking would not be viewed in the same fashion. Others avoided the topic despite parents being aware (*n* = 2) and the majority felt their parents would disapprove because it is still a form of smoking (*n* = 11). (Table 3).

#### 4.2.3. Lack of Knowledge

- Waterpipe vs. Cigarettes

Current (*n* = 2) or previous (*n* = 2) cigarette smokers perceived waterpipe smoking to be a much more appealing form of smoking, due to the fruity flavours, smoother texture and being able to smoke for extended periods of time. All were convinced that they were not addicted to smoking waterpipe tobacco and could very easily give it up, despite some (*n* = 2) expressing cravings. Only two participants (non-cigarette smokers) considered WTS to be equivalent or more harmful than cigarette smoking, with most being unaware that it contained any tobacco or toxins (*n* = 10). Respondents attributed this to a lack of both knowledge and negative connotations typically associated with cigarettes (Table 4).

#### 4.2.4. Perceived Risk

- Health Perceptions

All participants lacked any knowledge about the health implications of waterpipes. Some believed that the water bowl filtered the smoke (*n* = 4). While none thought the fruit flavours made the waterpipe any healthier, it did help to convince them that it is less detrimental to their health than other intoxicants. When asked about sharing mouthpieces, some would readily change the disposable plastic “tips” as the pipe is passed around (*n* = 4), whereas other felt this to be both impractical and rude (*n* = 12). None were able to describe any specific adverse effects when questioned. Concerns about personal health were low as the majority considered themselves to be infrequent smokers.

- Public Policy

None were aware of any public campaigns or policies aimed at controlling the use of WTS. Anti-smoking campaigns and public health interventions (from TV adverts, posters and leaflets to general practitioners or general dental practitioners inquiring about smoking habits) were perceived to be targeted at cigarette smoking. A dominant theme that emerged regarding regulation was the need for an age restriction on WTS. Some participants had witnessed teenagers in Shisha cafés being served (*n* = 2). Many expressed that educating the younger generations on the negative effects of WTS might result in fewer choosing to partake. Other suggested policies included increasing the cost of waterpipe tobacco (through a tobacco tax), limiting the number of Shisha cafés in any given area and statutory health warning and labelling (similar to that seen with cigarettes) (Table 5).

## 5. Discussion

To our knowledge, this is the first qualitative study exploring perceptions towards waterpipe tobacco smoking by young adults in the UK. The key themes that emerged all pointed to a lack of awareness of the harmful effects of WTS and rather its increased social acceptance in this cohort. Key attractions to the waterpipe included fruity flavours, pleasant odour, competitive pricing and the safe and conducive environment provided by cafés in which to smoke. This combination may be driving the rapid increase in waterpipe smoking in the UK, threatening to undermine the public health gains made over recent decades on reducing cigarette use. The relevance of these findings cannot be overlooked when trends are demonstrating that WTS may have overtaken cigarettes as the method of choice for smoking tobacco among the young adult population in the UK [3]. Urgent action is required to curb this growing trend and the associated morbidity and mortality that is likely to develop from the harmful effects of WTS.

WTS clearly has a strong social standing, with no associated taboo or stigma due to its inclusive nature and being perceived as significantly less harmful than cigarette smoking. This perceived lower risk to health can be attributed to a lack of public health information and the sensory cues of aromatic smells and fruit flavours [23], essentially masking the negative associations to tobacco smoking. Further, there also appeared to be element of denial, with all respondents considering themselves to be “infrequent smokers”, resulting in few having reason for concern. Worryingly non-smokers have also demonstrated a similar ignorance towards WTS, including a willingness to try WTS based on their harm beliefs [33].

Sharing the tip of the waterpipe between members of a group seems to be commonplace; some smokers do not mind, whereas others feel a sense of social pressure. As many as 92% have reported sharing a waterpipe hose among a group (3). Given the increase of oropharyngeal squamous cell cancer from transmission of HPV, this is a further health concern that is in danger of being disregarded [34].

The smoking environment of Shisha cafés has played a significant role in the increasing popularity of the waterpipe. The elaborately decorated terraces, fruity smells, and other amenities are attractive to customers. This commercialisation has resulted in WTS becoming a trend for the younger smoker. With the number of cafés opening not being regulated by the government, the UK has seen a 210% increase in the number of Shisha cafés in just five years [28]. As a result, access and affordability have increased, which are key drivers for uptake [24]. This is inadvertently sending out a negative message to the public that WTS is acceptable.

What is of particular concern is that there is an underground culture of Shisha cafés, which permit illegal smoking indoors. It has been demonstrated that the indoor air quality of Shisha cafés is far worse than a restaurant that permits cigarettes smoking [35], with high levels of carbon monoxide in the second-hand smoke. Further regulation is required to tackle this growing problem [36].

The evidence is inconclusive as to whether WTS is truly addictive in the same manner as cigarette smoking [37]. Our study found that smokers are drawn to the social dimension and environment than the smoking itself. Therefore, within this cohort it appears to be a social addiction. None of the participants felt they were addicted to the tobacco or nicotine and were able to willingly give the habit up should they wish. Finding an alternative social activity would be the struggle, however, especially for the smokers’ who do not drink alcohol or indulge in the “clubbing” scene. One respondent went as far to express a desire to quit WTS but felt his relationship with his shisha smoking friends would break down if he did not participate.

Central to the social acceptance of the waterpipe is the lack of knowledge about its harmful effects. None could name specific diseases that might result from smoking and many were unaware that “maassel” contained any tobacco at all, thinking it to be simply “flavoured air”. The historic misconception that the water bowl acts as a filter removing any toxins from the smoke still exists. There is a clear need for providing information to the public about the harmful health effects so that they are able to make an informed decision regarding WTS. The challenge will be changing the behaviours of communities who have a cultural link to the waterpipe, where it has a high standing [23].

Previous studies have shown an increase in global WTS use [38], including increases in the student population [3,4,14,17,18,23,39]. Qualitative studies within student populations have also found similar findings to our study; however we also found that the social appeal of WTS continues beyond the university setting, with Shisha cafés playing a key role in its increasing popularity [3,4,14,17,18,23,39]. Further, qualitative studies that have primarily taken place within the student population demonstrate similar findings to our study; but the current study shows that the social appeal of WTS continues beyond the university setting, with Shisha cafés playing a key role in its increase in popularity. London is a global metropole, and results from this study may be relevant for other large, cosmopolitan cities; findings from studies in similar settings in other countries are broadly similar [13,15,16,17,18,19,20,23,24,34], identifying a growing need for public health interventions on a global scale.

## 6. Strengths and Weaknesses of the Study

A limitation was that only one researcher conducted the interviews and coding. That said, the researcher adopted a participant observer role and we triangulated the findings as far as possible by having two authors (AD and VA) listen to the recordings, followed by a discussion.

Despite this limitation, the study had a number of strengths. First, data was independently transcribed and the accuracy of the transcriptions was confirmed, by member checking, increasing internal validity. Second, participants verified the researcher’s emerging themes and inferences generated from the interview dialogues, maximising credibility. Third, recruitment itself took place at different times of the day to ensure that both day and evening smokers were included (environmental triangulation). Themes remained consistent across the locations. Fourth, purposive sampling allowed for a variation in demographics in particular ethnicity of waterpipe smokers. Females were more responsive than men with a ratio of 10:6. However, no gender differences in themes emerged. Fifth, the researcher kept an audit trail discussing discrepancies with VA and developed interviews and topic guides through constant comparison thus maximising dependability and adding to confirmability.

## 7. Future Research

Future research should aim to separate the smokers into the two groups; the Shisha café users and the home smokers. It is plausible that each cohort of smokers holds different views. Our study was not able to assess the views of home smokers due to insufficient numbers. The perceptions of all stakeholders (i.e., Shisha café and restaurant owners) should be sought to adequately formulate prevention strategies and further understand the surge in popularity and trends in advertisement.

With no infection control regulation in Shisha cafés, and sharing of mouth-pieces being common practice, transmission rates of HPV which can lead to oropharyngeal carcinoma and respiratory virus such as COVID-19 is a hazard and should be further investigated.

Previous research suggests that WTS may be addictive but further investigation is required. We have established that most report a social addiction but could this be masking a “true” nicotine addiction, particularly in frequent smokers. The use of e-cigarettes is also increasing, and this growing trend is likely to affect the future use of WTS and should be considered in future research on WTS use.

## 8. Clinical Implications

The findings of the study have implications for public health policy. The dimensions identified (lack of knowledge, perceived risk, affordability and social addiction) all point towards an increasing acceptance of WTS among this cohort and therefore a brewing public health risk related to potentially increased morbidity and mortality in this group; not only from direct effects of tobacco but also from second hand smoke and the transmissions of infectious diseases like HPV that can lead to more fatal oropharyngeal cancers.

Typically, waterpipe smokers do not identify themselves as smokers, and therefore this group of tobacco users are unidentifiable to healthcare professionals [40]. The lack of factual information of the adverse health effects associated with WTS has allowed the perception that it is healthier than other forms of smoking to prevail and thus, smokers have no reason to stop its use. Therefore, taxation of maassel [41], age restrictions, and statutory health warnings on the packaging or apparatus [42] might be useful starting points to inform future cessation policies. However, these interventions will need to be tailored depending on the various motivations for WTS (e.g., cultural tradition vs. social activity).

## 9. Conclusions

This study identified important drivers of WTS, particularly related to affordability, perceived lower risk and social addiction, especially among those who do not drink alcohol. The recent increase in the use of WTS in combination with the drivers we have identified is a public health threat with the potential to generate substantial increases in morbidity and mortality from the harmful effects of WTS. Accurate and targeted prevention is important, but must be accompanied by strategies that recognise the strong social standing and cultural motivations associated with waterpipe use, in addition to the adverse health effects.

## Figures and Tables

**Table 1:** Demographics of Waterpipe Smokers Interviewed.

	Total
Gender	
Male	6 (37.5%)
Female	10 (62.5%)
Age Group	
20–25	9 (56.25%)
26–30	7 (43.75%)
Ethnicity	
Asian	5 (31.25%)
Arab	6 (37.5%)
White	3 (18.75%)
Other	2 (12.5%)
Smoking	
Café	13 (81.25%)
Home	0
Both	3 (18.75%)
Initiation	
Pre-University	1 (6.25%)
At University	14 (87.5%)
After University	1 (6.25%)
Other Tobacco use	
Yes	2 (12.5%)
No	14 (87.5%)

**Table 2:** Quotes belonging to the dimension ‘availability and affordability’.

Theme	Corresponding Quote(s)
Availability & affordability	“It’s the ease of availability. You can get hold of shisha pretty much anywhere. There is a corner shop near my house where you can buy it pretty cheaply.” (T4)
	“They are erm, everywhere you look. I live in an area with a high Arab population and Shisha Cafes are everywhere to be seen. Some people like to even do it while they eat, similar to back in the Middle East.” (T6)
	“Yeah, I guess I smoke less because of it [Not having a Shisha Café nearby]. When I’m visit my friends who do live near one I can pretty much bet that is where we will be heading.” (T2)
	“I think if it was really far away, it would really make a difference as it can be difficult to go after work. I actually have one around a five-minute drive from my house … I think it is unlikely that I would stop unless I moved to a place where it is not readily available.” (P7)
	“And its cheap cos the cost gets divided by the whole group. I mean to sit in one place for a few quid for a good few hours ain’t that bad. Bars cost so much more.” (T4)
	“Last time we went between a group of 3 of us we paid £10 each including a drink. And that shisha will last for two hours or more. £10 for 3 people for 2 h, that’s nothing really.” (P9)
	“But in the UK it’s become better known. There are so many cafes making it a trendy thing to do. It’s a cheap thing to do.” (T5)
	“It’s also advertised too much. A lot of shisha gardens are front of house so you walk by and you smell it. So everyone can see and smell it. Its more tempting then.” (P5)
	“I think if shisha did not have a flavour it’s unlikely that I would do it. I like the fact that when you just walk by erm a café the kinda sense almost calls you in. And that smell surrounds the area.” (P7)

**Table 3:** Quotes belonging to the dimension “social addiction”.

Theme	Corresponding Quote(s)
Social appeal	“Smoking alone would make me think that I was addicted to it, which I don’t think I am. And err rocking up to a Shisha Café alone err like no one really does that … you would always go with friends.” (P7)
	“It is most definitely a group thing that you would do with your friends or cousins. I can’t think of anyone that would just do it by him or herself. It’s just great to sit in the atmosphere with friends chilling and catching up.” (T7)
	“It’s got such a massive cultural and social aspect … I don’t think there is any harm in just doing nothing at home and having some shisha. So I would smoke by myself at home.” (T5)
	“I think it’s addictive among social situations. Like you always do it with your friends in happy situations … I don’t think it’s just shisha that is addictive; it’s the whole social situation. Like you’re with your friends, you’re having a good time. The café is kinda like a bar with alcohol, so it could be the situation that is addictive rather than shisha itself … Its similar how people have a glass of wine to unwind.” (P4)
	“The main reason for smoking is socially. It’s very much a social activity for me. I would compare it to going out for drinks.” (P7)
	“It’s more about socialising. I don’t like doing it but it’s err something quite common and acceptable in my kinda religion so err it’s a socialising thing. I don’t drink alcohol and you can only go for so many ‘coffees’ so shisha is a good alternative … Cos there is not really a lot to do. So, say if you’re with your other half, apart from cinema and you don’t go clubbing or drink, shisha is the one thing you can do together and its acceptable. So even if you don’t wanna do it sometimes, it just ends up being something that you do.” (T2)
	“And some of my friends don’t drink alcohol so that’s more what they do [waterpipe smoking] … because it gives me a headache. So I basically do it to fit in.” (P3)
	“Definitely a group activity. It’s not something I would ever consider doing on my own. I usually pretty much always smoke with friends … and doesn’t have the nasty err side effects of a clubbing alcohol fuelled night out. So the next day you can just carry on with work and stuff … Your still in control, you still know who you are and where your are and stuff.” (T4)
Environment	“Shisha cafes provide the ultimate experience really to go along with the smoking itself. I find that I’m drawn to those cafes that have the elaborately decorated terraces, reminding me of holidays to places like Morocco.” (T7)
	“The cafes usually have quite nice atmospheres, they are quite comfortable, quite warm, with lanterns and sofas. It has a strong Mediterranean feel that makes you feel like you’re away on holiday in Morocco or the Middle East. It makes you feel very cultural as well … thinking about it now, I don’t know if it is the shisha or the environment with all the decorations and music which relaxes me more.” (P7)
	“We did it at home mainly cos there was nowhere else to err do it. Before the cafes use to be so unwelcoming and dark and ugly. Now the first choice to smoke would be a nicely decorated café … The Shisha lounges kinda become our places to just meet up and chill. And cos we go all the time it’s just easy. No one makes a fuss or effort. It’s just like being in my own lounge [laughs].” (T3)
	“It is less in winter as we are smoking outside, however a lot of the cafes have a warm atmosphere and there are heaters everywhere. And it’s kinda nice actually to wrap up warm, sit near a heater and smoke shisha. So I’d say it is less but I do still go.” (P7)
	“And also there are indoor shisha places as well.” (P4)
	“Yes but I think that’s a hidden secret [indoor Shisha Cafes] and officially shisha is not meant to be done indoors.” (P1)
Cultural relevance & family acceptance	“It more of a cultural thing … That’s why in our culture people say its ok cos it’s not quite smoking … So, I think depending on what culture or cultural background you’re from and whether smoking is acceptable or not … It’s going to be hard to stop people who have it in their cultural background. We’re talking about Asian and Middle Eastern people … A ban or restriction may be difficult for them to understand.” (P5)
	“My friends from other cultures it has quite a strong standing, especially people from a Middle Eastern backgrounds. Cos those are my friends that introduced me to it in the first place … cos they don’t drink alcohol, so for them it is acceptable and a good thing to do.” (P8)
	“It’s very big in my culture and very big in my community. Literally, I’ve grown up with it … drinking is forbidden and then, I feel the youth need to have something they can do. And this is basically it … It’s more a positive thing. Celebrations, family gatherings, social meet ups. It just gets everyone together.” (T5)
	“Obviously coming from a background where it is encouraged I don’t feel the need to hide it from my parents. But they wouldn’t like me smoking cigarettes really. That’s a whole different ball game [laughs]. My dad would give me so much stick for that. Shisha on the other hand is fine.” (T6)
	“I did [own a waterpipe] but chucked it away because I only really smoked it in my flat at uni. After I left, I knew I wouldn’t do it at home around my family and parents … I don’t think my parents know that its tobacco. I honestly don’t think they even realise what it is. They think it’s just like a novelty thing and they’ve been to like Egypt and they think it’s just one of these fun things to do.” (P9)

**Table 4:** Quotes belonging to dimension “lack of knowledge”.

Theme	Corresponding Quote(s)
Waterpipe vs. cigarettes	“You don’t smell like a smoker. You smell of somebody who errr nothing. It smells nice. And it tastes nice as well … The fact that it is flavoured makes it easier and we all smoke it more as a result. It’s like alco-pops; packaging a bad thing in a better medium. I don’t think they make it healthier [the flavours] but I would say I’m able to convince myself that it’s less harmful because it doesn’t taste as bad.” (P1)
	“It’s much easier to smoke shisha than smoking cigarettes. It’s much more enjoyable, it tastes better, it smells betters. It doesn’t give you the awful breath like you do after a cigarette.” (T4)
	“I think it’s seen as better than doing cigarettes or cigars, cos there is so much taboo around normal smoking and there is so much emphasis on the NHS about stopping smoking. All these adverts, phone lines and your doctor always asks you about it. Nobody ever asks you if you smoke shisha. I don’t think its seen as a problem or being that bad.” (P9)
	“It’s more of habit. I don’t feel like it does anything for you. In terms of intoxicating you at all. And it’s not even like an addiction. It’s more just a craving. You know like you crave specific food and things like that.” (T5)
	“I think it’s much better, than erm other forms of smoking. Because you hear about the repercussions of smoking stuff like cigarettes or weed. I have never heard anyone talking about shisha being bad.” (T5)
	“Not everyone is aware that it contains tobacco. I know many people that think it’s tobacco free … people don’t know the full extent of what’s actually in it. They just do it.” (P5)
	“When your growing up, you know cigarettes are bad for health and its dangerous. As is alcohol, as is drugs, class A drugs, whatever, the side effects, the harmful effects of them. Whereas, we don’t know enough about shisha … There is no statutory warning. For those reasons, I have been smoking it all these years, thinking ‘if it was that bad they would of stopped it’.” (P1)

**Table 5:** Quotes belonging to dimension “perceived risk”.

Theme	Corresponding Quote(s)
Health perceptions	“There is the water filter and stuff, so it doesn’t feel as if it’s as bad for you … there is probably less nasty stuff than you get in a cigarette.” (T4)
	“We thought it was just air and just flavouring. I didn’t even know it was tobacco. I had no idea. And it’s not something that they advertise it. And I was surprised when I did find out it was tobacco.” (P9)
	“The next day I sometimes cough depending on how much I smoked and almost feel like the back of throat is swollen. But that’s its really.” (T7)
	“I wake up the next day feeling disgusting. Dry mouth, headache … during smoking you feel light headed so it makes you want to keep going.” (P9)
	“You do get those end nozzles. But who really changes those. I don’t like that either. I know I shouldn’t do it and it isn’t that good for you, but when you’re in the environment you just give in.” (P5)
	“The worst thing is if you were in the street you wouldn’t share a can of coke with a random [everyone laughs] but you would happily sit down with people you have just met and share a shisha pipe. You don’t know what people have got.” (P6)
	“I do think it’s not the most hygienic when you really think about it. But when you’re in the atmosphere, having shisha, you don’t really care about anything thinks or its effects. You just concentrate on having shisha and having a good time with your friends.” (P2)
	“You go there and think I really wanna try that flavour so yeah I think it does. I do think that the fruit flavours can give impression that it is less damaging than we think … what the flavours do is to hide the “nasty” taste which would give the impression that the habit was bad for you.” (P5)
	“You think it’s good for you don’t you, when its strawberry or pineapple or minty. And they give you like a mint tea with it, and you think ‘oh I’m being really healthy’ [laugh].” (P9)
	“Believe it or not these flavours and fruits masks what’s actually behind and how bad it is for you. But the fruit is what got me going in the first place … they are always introducing new flavours. And it makes you want to try it … Even while I’m doing shisha at the places I begin to get weird headaches. But I carry on doing it cos I enjoy the flavour so much.” (P8)
	“I haven’t heard of any terrible things happening to people who smoke it. I mean like people in the countries where it came from have been doing it for years and they seem to be fine. We haven’t heard any shock horror stories [laughs].” (P7)
	“I just don’t see it as being that bad cos the amount of people that smoke and how much they smoke. And I’ve been smoking for a long time and I train at the gym and I haven’t seen any adverse effects on my health.” (T3)
	“It’s not something that hugely concerns me because, I think of myself as infrequent smoker … Like a social smoker so. I don’t really consider myself at high risk of those thing.” (T4)
Public policy	“All the emphasis appears to be on cigarettes and that’s probably why no takes shisha as seriously. Anything bad about it I have heard is just hear say from friends.” (T7)
	“Like even when you see your doctor they ask about cigarettes and alcohol intake but never ask if you smoke shisha. Which is important for young people like us. No one says it bad. Everyone only really talks about [cigarette] smoking.” (P3)
	“Until there are negative impacts of shisha in the public eye, people won’t know about it and take proactive measures, like they do for smoking … But no one knows the health negatives of shisha, so why would they stop?” (P6)
	“It’s quite easy to just go into a shop and buy a shisha pipe. Like there is no restriction. But there are some places in London where you have to be 21 to get into the café. But they are quite flexible because if you look a certain age they still let you in.” (P4)
	“I personally think there should be an age restriction because I think if young children start smoking shisha it could potentially lead to trying other things.” (T2)
	“And it is too affordable and accessible, especially to children. Like cigarette packets have laws on them. I think there should be an age restriction on shisha.” (P3)
	“If it is 10 times stronger than smoking surely the government should raise the price of shisha as they have with cigarettes in accordance with the amount of tobacco it contains.” (P6)
	“I think I would find it very interesting if they came up with a scenario where it was really really harmful to my health. I’m not aware that it is and I don’t think it is … And it is advertised in such a positive fashion almost luring people in. Advertising affects how you view a product and it will affect how I buy it. So marketing and advertising has a really big effect. So if that marketing and advertising was negative it was affect uptake of shisha.” (P7)

## Supplementary Materials

The following are available online at https://www.mdpi.com/2304-6767/8/4/134/s1, Figure S1: Questionnaire on Smoking Habits, Figure S2: Topic Guide for a Focus Group Discussion with Shisha Pipe Smokers.
