# Supplementary material for: Perceptions of Waterpipe Smoking among Young Adults: A Phenomenological Study
Source: Dent J (Basel). 2020 Dec 10;8(4):134. doi: 10.3390/dj8040134 (PMC7763057; doi:10.3390/dj8040134)
Supplement: Supplementary file 1 [file dentistry-08-00134-s001.pdf]

Supplementary Figure 1:
Questionnaire on Smoking Habits

Thank you for taking the time to complete this questionnaire. There is no right or wrong answer to answer to any of questions.

Year of Birth ____________________________________

Gender Male ☐ Female ☐

Nationality ____________________________________

Ethnicity____________________________________

Smoking Habits
How long have you been smoking for? __________________

Number of tobacco products smoked –
On average how many of the following products do you smoke?

1. Manufactured cigarettes?...per day
2. Hand-rolled cigarettes?...per day
3. Pipes full of tobacco?..per day
4. Cigars?...per day
5. Number of waterpipe/ shisha sessions……………………….....per week

   Duration of shisha session on average……….……………………..hours
6. Other?...

First Experience of Shisha -
Where did you start smoking Shisha?

1. Before University………………………………………………….. ☐
2. During University…………………………………………………….. ☐
3. After University……………………………………………………….. ☐

Preferred location to smoke Shisha –
Where do you tend to smoke the most?

1. Café………………………………………………………………………….. ☐
2. Home……………………………………………………………………….... ☐
3. Other…………………………………………………………………………. ☐

How often do you or someone you live with smoke at Shisha at home?

1. Daily………………………………………………………………………….. ☐
2. Weekly………………………………………………………………………. ☐
3. Monthly……………………………………………………………………. ☐
4. Less than monthly…………………………………………………….. ☐
5. Never……………………………………………………………………….. ☐
6. Don't know…………………………………………………………………. ☐

Receiving Cessation Advice from a Doctor –
During a visit to a doctor or dentist, did they enquire about whether you smoke Shisha?

1. Yes…………………………………………………………………………..... ☐
2. No……………………………………………………………………………... ☐

Noticing any Anti-Waterpipe/ Shisha Information in Media –
Have you ever noticed information about the dangers of Shisha smoking or any media that encourages quitting?

4. Yes……………………………………………………………………………. ☐
5. No…………………………………………………………………………….. ☐

Adapted from World Health Organisation, Global Tobacco Surveillance System, A subset of Key Questions from the Adult Tobacco Survey. 2011

Supplementary figure 2:
Topic Guide for a Focus Group Discussion with Shisha Pipe Smokers

INTRODUCTION

1. Facilitator: *"Hello, my name is XXXX, from Queen Mary University of London Dental Institute and thank you coming along today to take part in this discussion. The purpose of this discussion is to understand why people smoke shisha as it is growing trend in the Western world. Hopefully by identifying why people smoke shisha, effective methods can be introduced to address this new emerging habit"*
2. Ask participants to introduce themselves for transcription purposes
3. Explain the purpose of the interview and the ground rules:
    a. *Time constraints – "Since we have limited time, I'll ask that questions or comments off the topic be answered after the focus group session"*
    b. *Equal participations – "I'd like to hear everyone speak so I might ask people who have not spoken to comment"*
    c. *Mutual respect – "Please respect each other's opinions. There's no right or wrong answer to the questions I will ask. We want to hear what each of you think and it's okay to have different opinions"*
    d. *Confidentiality – "We want to keep the sessions confidential so we ask that you not use names or anything directly identifying when you talk about your personal experiences. We also ask that you not discuss other participants' responses outside of the discussion. However, because this is in a group setting, the other individuals participating will know your response to the questions and we cannot guarantee that they will not discuss your responses outside of the focus group"*
4. Explain the format of the focus groups, audiotaping and verbatim transcription
5. Indicate how long the focus group will take (60 minutes)
6. Allow participants to clarify any doubts about the interview

SECTION 1: Smoking Habits

1. To get started, tell me little about how often you smoke shisha?
    - *Probe:* Is the presence of shisha smoking facilitates nearby a contributing factor?
    - *Probe:* Do many of you own a shisha pipe at home?

2. Where do you smoke shisha more regularly?
    - *Probe: What factors contribute do how often you smoke? Cost? Location?*
    - *Probe: Do you find during winter months you are less inclined to go to a Café?*

3. Who, if anyone, do you tend to smoke with? Do you smoke regularly by yourself? Is it regarded as individual or group activity?

SECTION 2: Views on Smoking

1. What do you think about shisha smoking? What do other think about shisha smoking?
    - *Probe: Family, friends, parents?*
    - *Probe:* Do you feel the flavours of shisha influence your opinion?

2. Do you think shisha smoking has any effects on your body?
    - *Probe:* Do you feel it's better or worse than other types of tobacco use?
    - *Probe:* Are you happy sharing the pipe with other people?
    - *Probe:* What makes it a better or worse form of tobacco smoking?

3. Is shisha smoking acceptable within your religion?
    - *Probe: Do you feel it is a intoxication?*

SECTION 3: Reasons for Smoking/ Level of addiction

1. Why do you tend to smoke?
    - *Probe:* Cultural relevance?
    - *Probe:* Social activity?
    - *Probe:* Stress related? To relax?

2. Are there any specific triggers that you would shisha smoke?
    - *Probe:* Stress? Work? Studies? Family?

3. Do you feel that you are able to stop smoking shisha?
    - *Probe:* Do you experience cravings for shisha smoking?

SECTION 4: Public policy/ Media

4. Have you seen any anti-shisha smoking campaigns? Adverts? Leaflets?
    - *Probe:* Is information readily available about what shisha can do?

5. Would accurate information change your opinions on shisha and even make you considering stopping?
    - *Probe:* Religious input?
    - *Probe:* Medical consequences?
    - *Probe:* Information in cafes?
